# Passive leg raising for predicting fluid responsiveness: a systematic review and meta-analysis

**DOI:** 10.1186/2197-425X-3-S1-A587

**Published:** 2015-10-01

**Authors:** X Monnet, J-L Teboul

**Affiliations:** Paris-South University Hospitals, Paris-South University, Medical Intensive Care Unit, Inserm UMR_S 999, Le Kremlin-Bicêtre, France

## Introduction

The passive leg raising (PLR) test has been proposed to predict fluid responsiveness in patients with acute circulatory failure. We reviewed and meta-analysed the studies that investigated the PLR-induced changes in cardiac output (CO) or surrogates and the PLR-induced changes in arterial pulse pressure (PP) as predictors of fluid responsiveness.

## Methods

MEDLINE, EMBASE and Cochrane Database of Systematic Reviews were screened for relevant original and review article. Studies quality was assessed with the QUADAS-2 scale.

## Results

Twenty-two studies including a total of 975 patients responded to the selection criteria for the PLR-induced changes in CO. In these studies, CO was measured by echocardiography in 7 studies, transpulmonary thermodilution in 7 studies, bioreactance in 4 studies, oesophageal Doppler in 3 studies, pulmonary artery catheter in one study and suprasternal Doppler in one study. Among those 22 studies, 9 including a total of 432 patients responded to the selection criteria for the PLR-induced changes in PP. Data are reported as mean (95% confidence interval) or mean ± standard deviation. All studies but two were conducted in adults. The pooled correlation between the PLR-induced changes in CO and the volume expansion-induced changes in CO was 0.73 (0.69-0.77). For the PLR-induced changes in CO, the pooled sensitivity was 0.81 (0.84-0.87) and the pooled specificity was 0.90 (0.87-0.92). The pooled area under the Receiver Operating Characteristics (ROC) curve was 0.95 ± 0.01. The mean of the best threshold was a PLR-induced increase in CO of more than 10 ± 2%. For the PLR-induced changes in PP, the pooled sensitivity was 0.51 (0.44-0.58) and the pooled specificity was 0.84 (0.78-0.89). The pooled area under the ROC curve was 0.79 ± 0.04 (p < 0.01 vs. the area under the ROC curve for the PLR-induced changes in CO). The mean of the best threshold was a PLR-induced increase in PP of more than 12 ± 4%.

## Conclusions

The PLR test is highly reliable in predicting the response of CO to volume expansion in patients with acute circulatory failure. Its predictive value is significantly better when its effects are assessed by measuring CO or a surrogate of CO than when measuring PP, in particular with a lower specificity.

